# Apoptotic efficacies of AgNPs formulated by *Syzygium aromaticum* leaf extract on 32D-FLT3-ITD human leukemia cell line with PI3K/AKT/mTOR signaling pathway

**DOI:** 10.1515/biol-2025-1161

**Published:** 2025-09-23

**Authors:** Liang Guo, Ru Kou, Guang Li, Yanping Song, Yunjie Zhang

**Affiliations:** Hematology Experimental Center, Institute of Hematology, Xi’an Central Hospital, Xi’an, 710003, China; Laboratory Department, Xi’an Central Hospital, Xi’an, 710003, China; Institute of Hematology, Xi’an Central Hospital, Xi’an, 710003, China

**Keywords:** silver nanoparticles, *Syzygium aromaticum* leaf, leukemia, 32D-FLT3-ITD, PI3K/AKT/mTOR

## Abstract

Clove, *Syzygium aromaticum*, is a medicinal plant from the Myrtaceae family with various applications in traditional medicine. The plant has been studied for its analgesic, anti-inflammatory, antiviral, and anticancer properties. This study focuses on the green synthesis of silver nanoparticles using clove leaf extract. The synthesized NPs were characterized using chemical methods and their anticancer activity was tested against a leukemia cell line, along with the signaling pathway that followed. The AgNPs were synthesized in a spherical shape and were less than 50 nm in size. The cytotoxic effects of the AgNPs on PCS-800-011 primary peripheral blood mononuclear cells and 32D-FLT3-ITD leukemia cells were evaluated over 48 h using the 3-(4,5-dimethylthiazol-2-yl)-2,5-diphenyltetrazolium bromide assay. The cancer cells showed reduced viability with an IC_50_ value of 162 µg/mL after exposure to the AgNPs. Through a detailed examination of the mTOR pathway, it was observed that AgNPs can alter the phosphatidylinositol 3-kinase/protein kinase B/mammalian target of rapamycin pathway, affecting 32D-FLT3-ITD cell growth and death. This pathway may contribute to the inhibition of the cell cycle and induction of apoptosis by AgNPs. For this reason, AgNPs may be used as a natural anti-cancer treatment for leukemia.

## Graphical abstract

**Figure:**
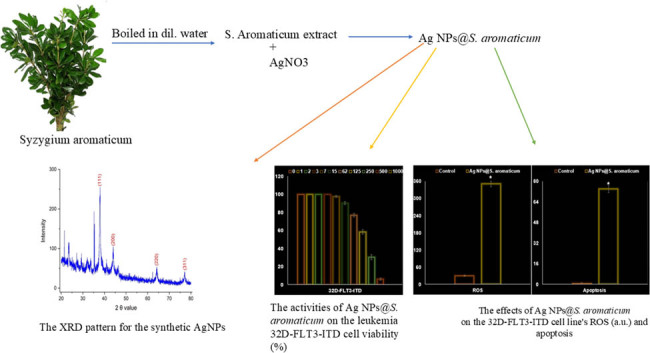

## 1 Introduction

Nanotechnology is one of the most groundbreaking developments of the twenty-first century. This field involves the transdisciplinary synthesis, management, and application of materials smaller than 100 nm. Nanoparticles (NPs), the products of nanotechnology are widely used in various fields such as technology, health, food, agriculture, environment, biotechnology, biomedicine, and medicine [1–3]. NPs are increasingly recognized for their potential in therapeutic applications, particularly in targeting specific patient cells or abnormal tissues through both passive and active targeting mechanisms. Various innovative strategies employing NPs have been developed, with a significant focus on addressing tumor heterogeneity and the tumor microenvironment. This has provided valuable insights for clinicians and nanotechnologists working toward the advancement of targeted drug delivery systems aimed at abnormal cells [4–6]. The investigation of modern NPs as a cohesive platform in treatment strategies aims to enhance effectiveness, safety, biocompatibility, and specificity while reducing toxicity and addressing the limitations of conventional chemotherapy. The integration of NPs into therapeutic approaches holds the potential to overcome major challenges associated with traditional treatments [5–8]. Silver nanoparticles (AgNPs) are being increasingly used in oncology for therapeutic and diagnostic purposes, providing novel strategies for cancer treatment. Their uses range from acting as nanocarriers for targeted delivery of chemotherapeutic agents to improving the effectiveness of photodynamic therapy and radiation therapy [9–11]. AgNPs have gained significant attention in the fields of diagnostics and research due to their various therapeutic applications. Recent studies have highlighted their potential cardiovascular protective effects, in addition to their established roles in cancer therapy [7–10]. Numerous research projects are currently focused on developing NPs as a viable option for designing drugs that can selectively target abnormal cells. This approach aims to enhance the efficacy of treatments while minimizing side effects commonly associated with traditional therapies [7–9]. Research indicates that AgNPs can effectively trigger apoptosis and sensitize abnormal cells, making them a promising candidate in cancer therapy. The effects of AgNPs are mediated through several mechanisms, including changes in cell morphology, decreased metabolic activity and lifespan, and the induction of oxidative stress. Reactive oxygen species (ROS) generation rises and mitochondrial damage results from these activities. These changes ultimately result in DNA damage, contributing to cell death [9–11].

Since ancient times, people from all civilizations and cultures have used medicinal and fragrant plants for various purposes such as nutrition, cosmetics, religion, therapy, and beautification. Additionally, they are used in nutrition as condiments, flavorings, herbal teas, and dietary supplements. These plants have a wide range of applications in industries such as cosmetics, fragrances, and body care products [12–15]. Clove, scientifically known as *Syzygium aromaticum*, is a medicinal plant from the Myrtaceae family that has gained significant attention in traditional medicine. Native to the Maluku Islands (Moluccas) in Indonesia, cloves are the aromatic flower buds of an evergreen tree that can grow between 8 and 12 m tall. They are widely recognized for their culinary uses as well as their various pharmacological properties [16–18]. Clove is a notable medicinal plant recognized for its rich chemical composition, which includes a significant amount of volatile essential oil, tannins, caryophyllene, triterpenes, and various esters. These compounds contribute to its numerous therapeutic properties and applications in traditional medicine [18,19]. Clove is not only valued for its culinary uses but also for its extensive medicinal properties, largely attributed to its rich chemical composition. Among the notable constituents are various glycosides, including light alcohols, monoterpenoids, eugenol, isoeugenol, farnesol, sitosterol, nerolidol, and campesterol. These compounds contribute to the therapeutic benefits of clove oil, which is extracted from the flower buds of the plant [19]. Clove oil is recognized for its numerous therapeutic benefits, particularly in treating wounds, injuries, and the effects of insect bites, especially on sensitive skin. Its rich composition of bioactive compounds, including eugenol, contributes to its effectiveness in these applications [17,18]. Clove oil is widely recognized for its effectiveness in treating various skin conditions, particularly acne. Its antibacterial and antiseptic properties make it a valuable ingredient in anti-pimple formulations, especially for addressing purulent pimples [17–19]. The plant extract has been traditionally utilized as an analgesic in dentistry and is gaining recognition for its complex antioxidant compounds, which may contribute to its anticancer effects. Recent research underscores the multifaceted benefits of clove, particularly its potent antioxidant properties and potential therapeutic applications [18,19].

Silver has shown promising potential for leukemia treatment due to its unique properties and advantages. In the present study, we focused on the green synthesis of AgNPs using the aqueous extract of the leaves of *S. aromaticum*, and evaluated the physio-chemical characteristics of the NPs using analytical methods. Furthermore, the application of NPs to prevent leukemia and cytotoxicity was evaluated by following the phosphatidylinositol 3-kinase/protein kinase B/mammalian target of rapamycin (PI3K/AKT/mTOR) signaling pathway. The PI3K/AKT/mTOR pathway is frequently overactive in cancer cells, resulting in unchecked cell survival, growth, and proliferation [20]. This pathway is essential in multiple facets of cancer progression, such as the formation of new blood vessels, the spread of cancer to other parts of the body, and the ability to withstand treatments [21]. Malfunctions in elements of this pathway, including excessive activity of PI3K, increased activity of AKT, and loss of mTOR function, are well-known contributors to treatment resistance and the advancement of cancer. Understanding the disruption of this pathway is crucial for creating successful cancer therapies [20,21].

## 2 Experimental

### 2.1 Green formulation of AgNPs

According to the principles of green chemistry, water is considered the best solvent for reactions due to its environmentally friendly nature and low cost. Additionally, plant aqueous extract are commonly used because they contain diverse array of natural biomolecules, such as flavonoids, phenolics, terpenoids, alkaloids, polyphenols, proteins, and carbohydrates which play essential roles in the synthesis process [22,23]. To demonstrate this, 10 g of dried leaves from *S. aromaticum* were ground and boiled in 150 mL of water for 10 min. After filtration, 20 mL of the resulting extract was combined with 100 mL of silver nitrate (10 mM) and stirred at a temperature of 50°C. After 24 h, AgNPs were produced. The precipitate was washed three times, centrifuged, and dried. The newly formed NPs were then subjected to characterization and used in biological experiments.

### 2.2 Chemical characterization

Several methods were used to characterize the produced AgNPs. A Shimadzu Fourier transform infrared (FT-IR) 8400 was used to capture the FT-IR spectra in the 400–4,000 cm^−1^ (KBr disc) range, while a Cary 50 was utilized to collect the UV–Vis spectrum (200–800 nm). The energy dispersive X-ray spectroscopy result and field emission scanning electron microscopy (FE-SEM) images were reported using MIRA3TESCAN-XMU. The transmission electron microscopy (TEM) image was recorded by a Philips EM 208S. A Stoe instrument was used to record the X-ray diffraction (XRD) pattern of MnNPs in the 2*θ* range of 20–80 at 40 kV and Cu-Kα radiation (1.5406 Å).

### 2.3 Evaluation of anti-leukemia properties

The 3-(4,5-dimethylthiazol-2-yl)-2,5-diphenyltetrazolium bromide (MTT) assay was used to evaluate the cytotoxic effects of AgNPs on both PCS-800-011 primary peripheral blood mononuclear cells and FLT3-ITD mutant 32D stable cell line (32D-FLT3-ITD) leukemia cells. Each type of cell was cultured separately in plates with 1 × 10^5^ cells per well. AgNPs were applied to the cells over 24 h at successive dosages ranging from 1 to 1,000 µg/mL. About 100 µL of 0.5 mg/mL MTT solvent (Product No. 88417, Sigma-Aldrich Company, USA) was added to each well to replace the medium for cytotoxicity assessment. After 4 h of dark incubation at 37°C, the medium was removed, and 0.1 mL of dimethyl sulfoxide was added to each well and mixed for 10 min. A microplate reader (Bio-Rad, CA, USA) was used to measure the absorbance at 570 nm. Cell viability was calculated using the following formula [24]:

\[{\mathrm{Cell\; viability}}(\left \% )=\frac{{\mathrm{Sample\; Absorption}}}{{\mathrm{Control\; Absorption}}}{\mathrm{\times }}100.]\]

The program GraphPad Prism version 9 was used to calculate the half-maximal inhibitory concentration (IC_50_) values. Additionally, a digital camera-equipped phase-contrast inverted microscope (Olympus, Japan) was used to observe the cellular morphology of both untreated and treated cells.

### 2.4 Molecular section of the study

The LDH test using cancer cells utilized the IC_50_ concentration. Following the instructions provided with the appropriate kit, membrane damage was assessed after treatment with NPs at the specified IC_50_ concentration. A spectrophotometer set to 450 nm was then used to measure the absorbance of NPs. As previously reported, 10^6^ PCS-800-011 and 32D-FLT3-ITD cells were used to measure ROS production using the DCFH2-DA staining test after incubation with NPs at IC_50_ concentrations and subsequent washing with PBS (Product No. BI-1401-05, Bio Idea Co., USA). The FITC probe (Product No. 33264, Cayman Chemical Company, USA) was used to detect apoptosis in PCS-800-011 and 32D-FLT3-ITD cells. A flow cytometer was then used to compare the percentage of apoptotic cells to the control group. One gram of RNA was used to create cDNA. The Applied Biosystems Step One Plus Real-Time PCR System (Product No. 4376592, Thermo Fisher Scientific Company, USA) was used in conjunction with the SYBR^®^ Premix Ex TaqTM II Kit (Product No. RR820A, Takara Bio Company, Japan) by the mentioned primers in Table 1 [24].

**Table 1:** List of forward (F) and reverse (R) primers sequences

Genes	Primer sequence
β-Actin	F-CCAACCGCGAGAAGATGA
	R-CCAGAGGCGTACAGGGATAG
Bcl2	F-TGGACAACCATGACCTTGGACAATCA
	R-TCCATCCTCCACCAGTGTTCCCATC
Bax	F-TTCATCCAGGATCGAGCAGG
	R-TGAGACACTCGCTCAGCTTC
Caspase-3	F-AGGACTCTAGACGGCATCCA
	R-CAGTGAGACTTGGTGCAGTGA
PI3K	F-CCACGACCATCATCAGGTGAA
	R-CCTCACGGAGGCATTCTAAAGT
AKT	F-TGAGGAGCGGGAGGAGTG
	R-GAGGATCTTCATGGCGTAGTAGC
mTOR	F-CAATGCTATGGAGGTTACAGG
	R-ATCGCTTGTTGCCTTTGG

### 2.5 Statistical analysis

SPSS-23 was used for data analysis, which included one-way ANOVA and a *post hoc* Dunnett’s multiple range test following a one-way analysis variance. The data were presented as mean ± standard error. The threshold for statistical significance was set at *p ≤* 0.01.

## 3 Results and discussion

### 3.1 Chemical characterization

**Figure 1:**
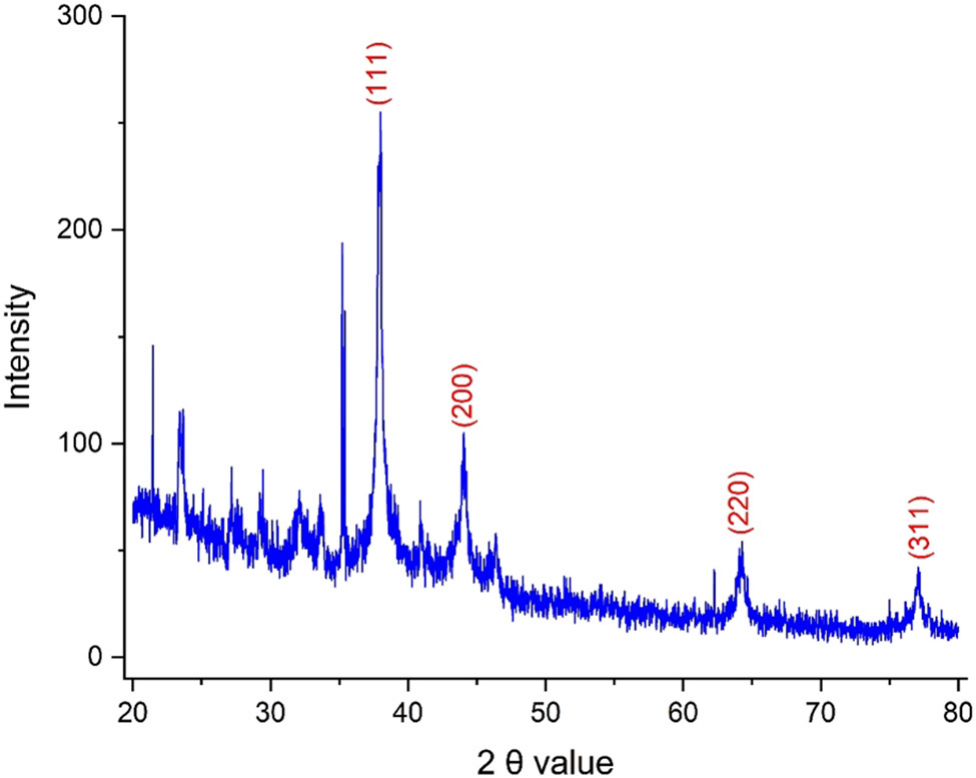
XRD pattern for the synthetic AgNPs using *S*. *aromaticum* extract.

Several analytical methods were utilized to characterize the produced AgNPs. The XRD pattern of the AgNPs is depicted in Figure 1, revealing their crystallinity. Significant peaks were observed at 2*θ* values of 37.835°, 44.180°, 64.205°, and 77.105°, corresponding to the crystalline planes (111), (200), (220), and (311). These results are consistent with the standard data from Joint Committee on Powder Diffraction Standards card (JCPDS card) 04-0783. The crystal size of the AgNPs was determined to be 30.03 nm using the Debye–Scherrer equation. Remarkably, these synthetic NPs demonstrated a smaller size compared to the crystal sizes reported for AgNPs that were green-synthesized using plant extracts such as *Gongronema latifolium* and *Berberis vulgaris* [25,26].

**Figure 2:**
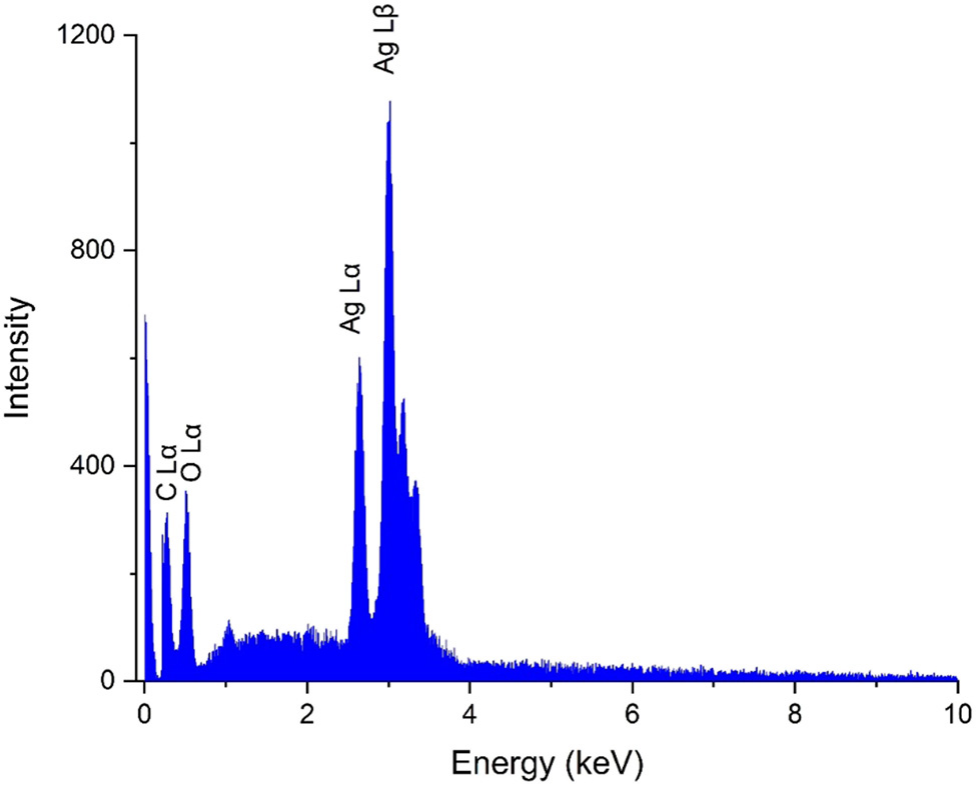
EDX diagram for the synthetic AgNPs using *S. aromaticum* extract.

**Figure 3:**
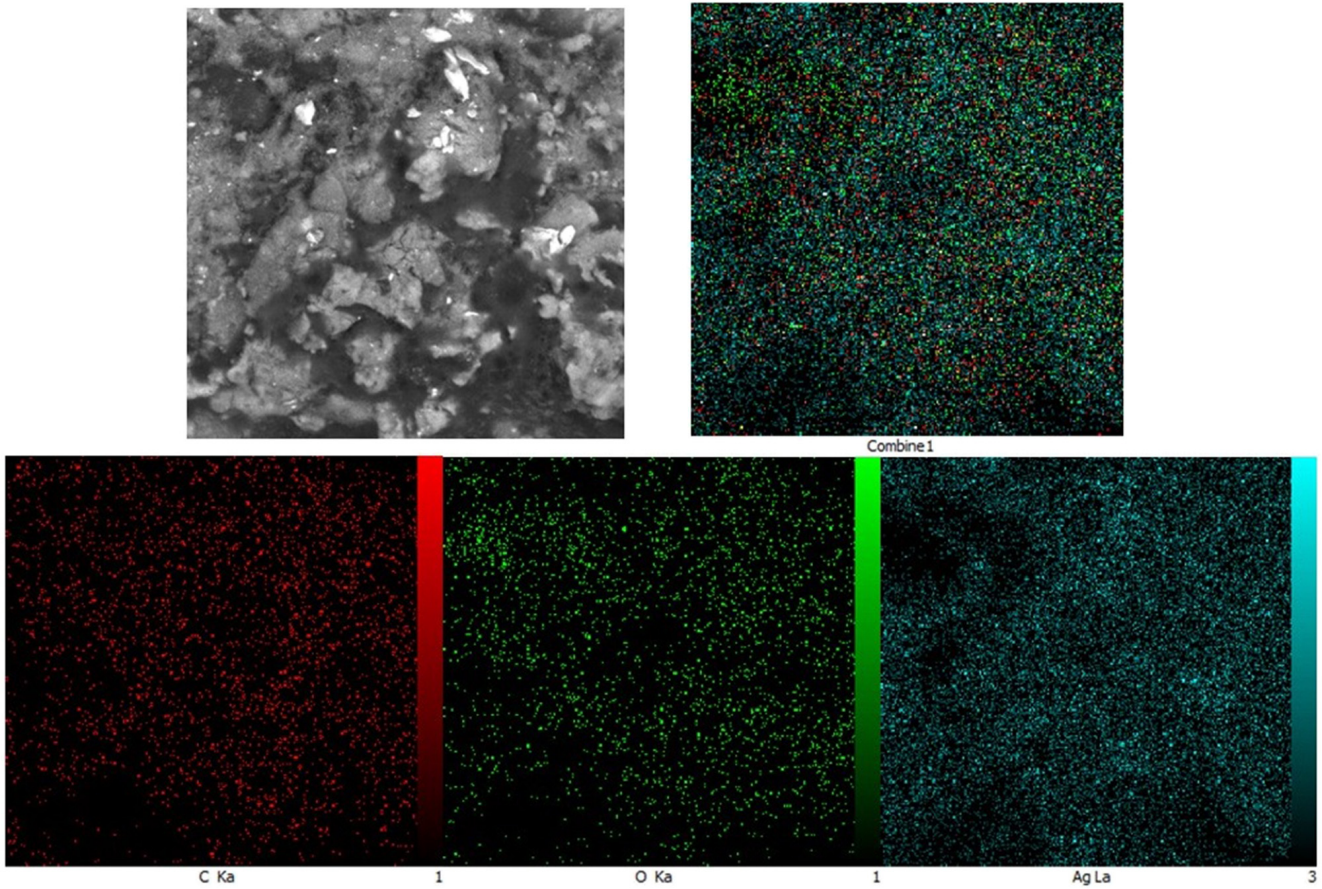
EDX mapping of the synthetic AgNPs using *S. aromaticum* extract.

Energy dispersive X-ray (EDX) analysis is an effective technique for elemental assessment of nanomaterials, providing detailed information about the elemental composition of the NPs. The EDX diagram reveals critical data about the NPs including confirmation of the target metal, elemental purity, and presence of other elements [27,28]. The EDX spectrum of AgNPs is shown in Figure 2, with peaks at energies of 3.02 and 2.64 keV corresponding to Ag Lβ and Ag Lα, respectively. Signals at 0.27 keV (C Lα) and 0.52 keV (O Lα) indicate the secondary metabolites binding from *S*. *aromaticum* to the surface of the synthesized AgNPs. Similar findings have been reported by Baghayeri et al., who observed comparable signals for AgNPs synthesized using *Salvia leriifolia* extract [29]. Moreover, by mapping the elemental distribution, EDX can help assess whether the metallic NPs are uniformly distributed and if the synthesis process yielded a consistent product revealing whether the metal and other elements (such as carbon and oxygen from plant biomolecules) are uniformly or heterogeneously spread [30,31]. The EDX mapping analysis in Figure 3, confirms the successful synthesis of AgNPs and the attachment of organic compounds to the NPs with a uniform distribution of elements of silver, oxygen, and carbon.

**Figure 4:**
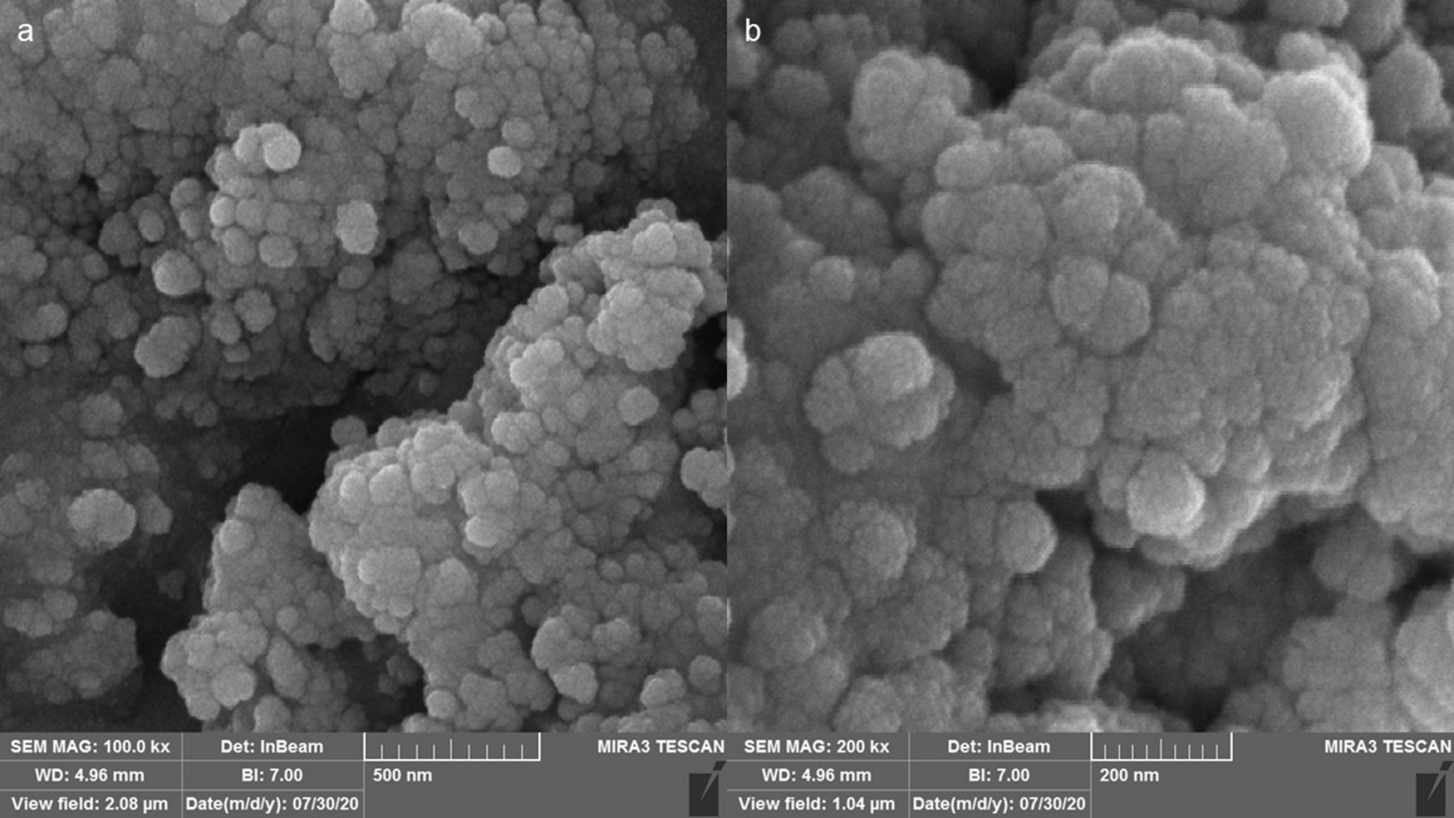
FE-SEM images of the synthetic AgNPs using *S. aromaticum* extract at different scales. (a) 500 nm; (b) 200 nm.

**Figure 5:**
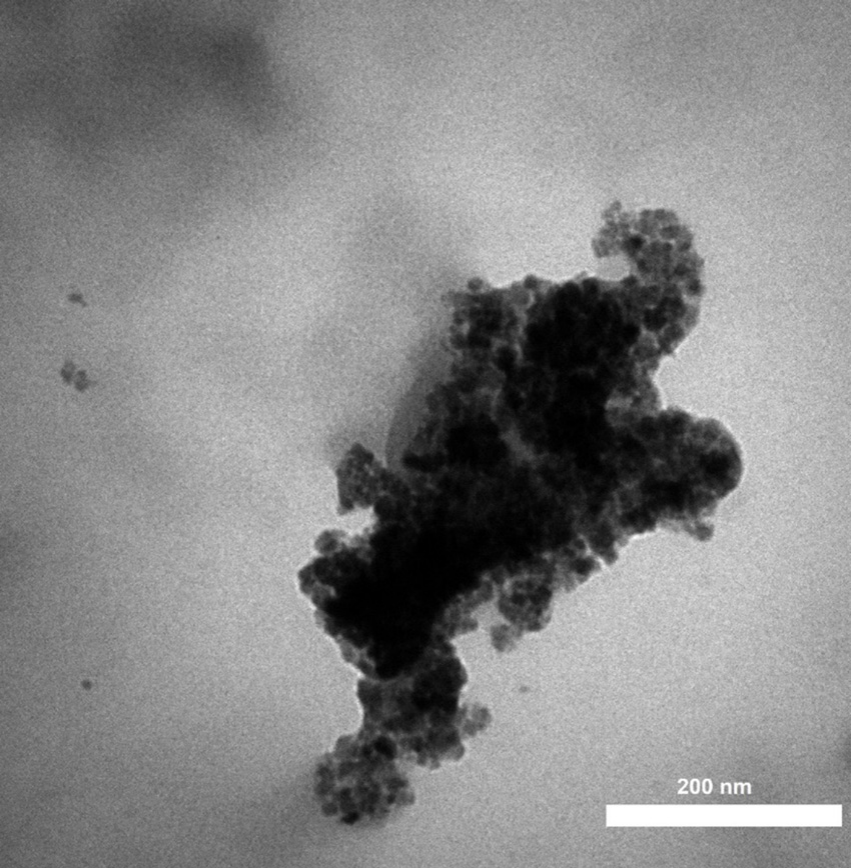
TEM image of the synthetic AgNPs using *S. aromaticum* extract.

Figure 4(a) and (b) presents the FE-SEM images of AgNPs. The AgNPs are completely formed in a spherical and aggregated morphology, which is a common physical property for AgNPs and other metallic NPs reported by many research groups [29,32–35]. The images show an average size of less than 50 nm for AgNPs in this study. Figure 5 displays the TEM image of AgNPs, which also reveals a spherical morphology and aggregation similar to the FE-SEM images. The size of the AgNPs in the TEM results was found to be the same as in the FE-SEM results. Both analyses show aggregation for the synthesized NPs. According to previous studies, aggregation usually leads to a decrease in biological activity because aggregated particles have less surface area and altered interactions with cells or microbes [36,37]. However, the AgNPs synthesized using *S*. *aromaticum* extract exhibited acceptable anticancer activity against 32D-FLT3-ITD cell.

**Figure 6:**
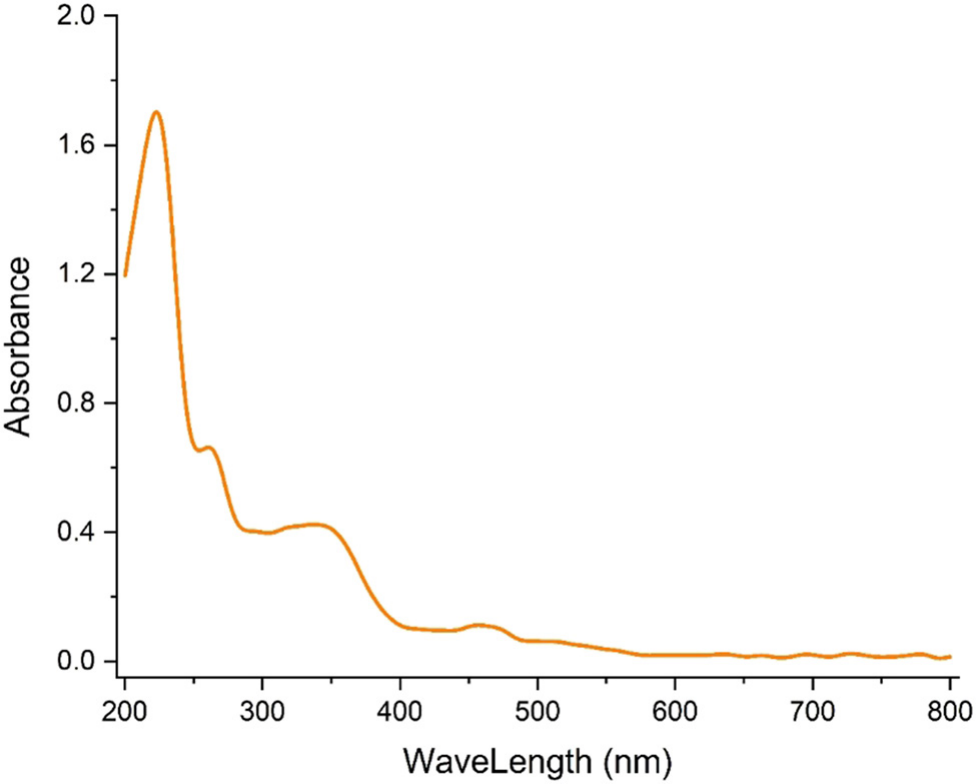
UV–visible spectrum of the synthetic AgNPs using *S*. *aromaticum* extract.

Surface plasmon resonance (SPR) is a phenomenon that occurs when photons excite the electrons on a metal surface. This phenomenon is investigated using UV–Vis spectroscopy, which is a sufficient technique to characterize metallic NPs [38]. The UV–visible spectrum of AgNPs is presented in Figure 6, with bands at 223, 260, and 346 nm indicating the successful formation of AgNPs. Renuka et al. reported bands at 235, 302, and 449 nm for the green synthetic NPs [39]. The primary cause of the variation in the wavelengths of the measured SPR for AgNPs is the reported NP size in the two investigations.

**Figure 7:**
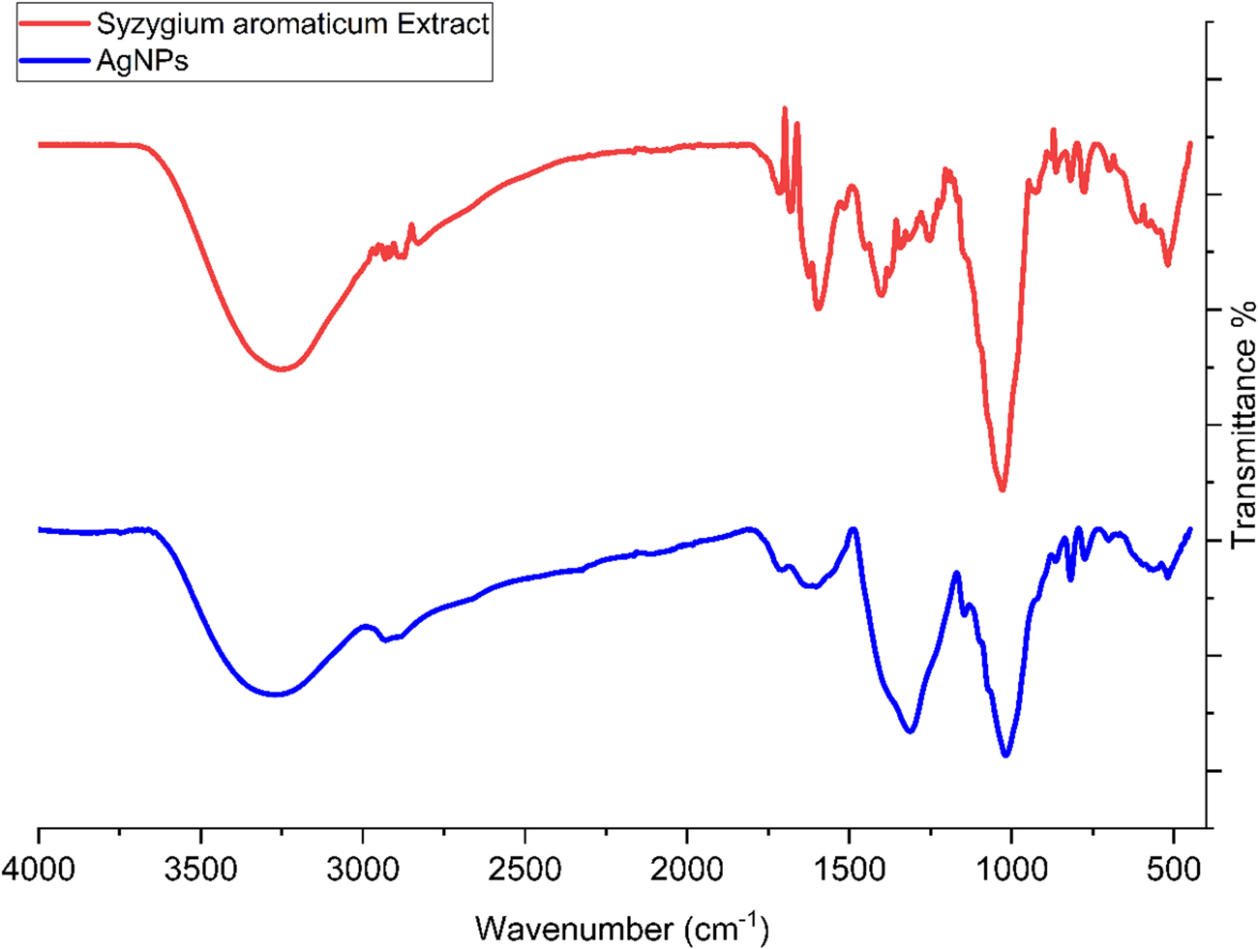
FT-IR spectrum of the synthetic AgNPs using *S*. *aromaticum* extract.

The FT-IR spectra of AgNPs and *S*. *aromaticum* extract are shown in Figure 7. The FT-IR method is a well-known qualitative approach for identifying metallic NPs. The FT-IR spectra of metal NPs synthesized using plant extracts reveal a close relationship between the functional groups present in the plant extract and the surface chemistry of the resulting NPs. This relationship is key to understand the synthesis mechanism, stabilization, and capping of the NPs. These compounds act as reducing agents that convert metal ions [40–42]. For metallic NPs, peaks in the range of 400–4,000 cm^−1^ are typically associated with metal bonds [32]. The peaks at 519 and 574 cm^−1^ are characteristic of AgNPs [24]. A comparison between the spectra of *S*. *aromaticum* extract and AgNPs reveals a similarity in the peaks of both spectra, confirming the linkage of the plant extract to the AgNPs surface. For example, bands such as those at 1,028, 1,603–1,714, 2,931, and 3,273 cm^−1^ are attributed to stretching vibration bands of C–O, C\C, C\O, C–H, and O–H, which are functional groups found in organic compounds such as flavonoids, terpenoids, phenolics, aldehydes, ketones, and other secondary metabolites that are abundant in *S*. *aromaticum* extract. The presence of these functional groups on the NP surface, as evidenced by FT-IR, implies that the phytochemicals form a capping layer around the NPs. This capping stabilizes the NPs by reducing agglomeration and providing biocompatibility [43,44]. These peaks often shift in position or change in intensity compared to the pure plant extract spectra, indicating interaction between the phytochemicals and the NP surface. Such shifts confirm that these functional groups are involved in the reduction and capping process. Thus, FT-IR spectral analysis clearly supports that the bioactive moieties of *S. aromaticum* leaf extract are preserved and play a key role in the formation and stabilization of the synthesized AgNPs.

### 3.2 Anti-leukemia effects of AgNPs

Recent studies have revealed that AgNPs exhibit a high degree of selectivity toward cancer cells, surpassing the therapeutic indices of certain commonly used chemotherapeutic drugs in animal studies. While metal-oxide NPs are considered safe (GRAS), their impact on cancer cells can range from benign to malignant. Through metal cation homeostasis, the metal ion can enter a normal cell and either inhibit cancer cell growth or serve as a supplement. Conversely, the remaining metal ions can act as antioxidants or ROS, respectively [24]. To assess the potential and safety for systemic use, it is crucial to conduct both *in vitro* and *in vivo* research on the cytotoxic effects of NPs produced using a green synthesis approach.

**Figure 8:**
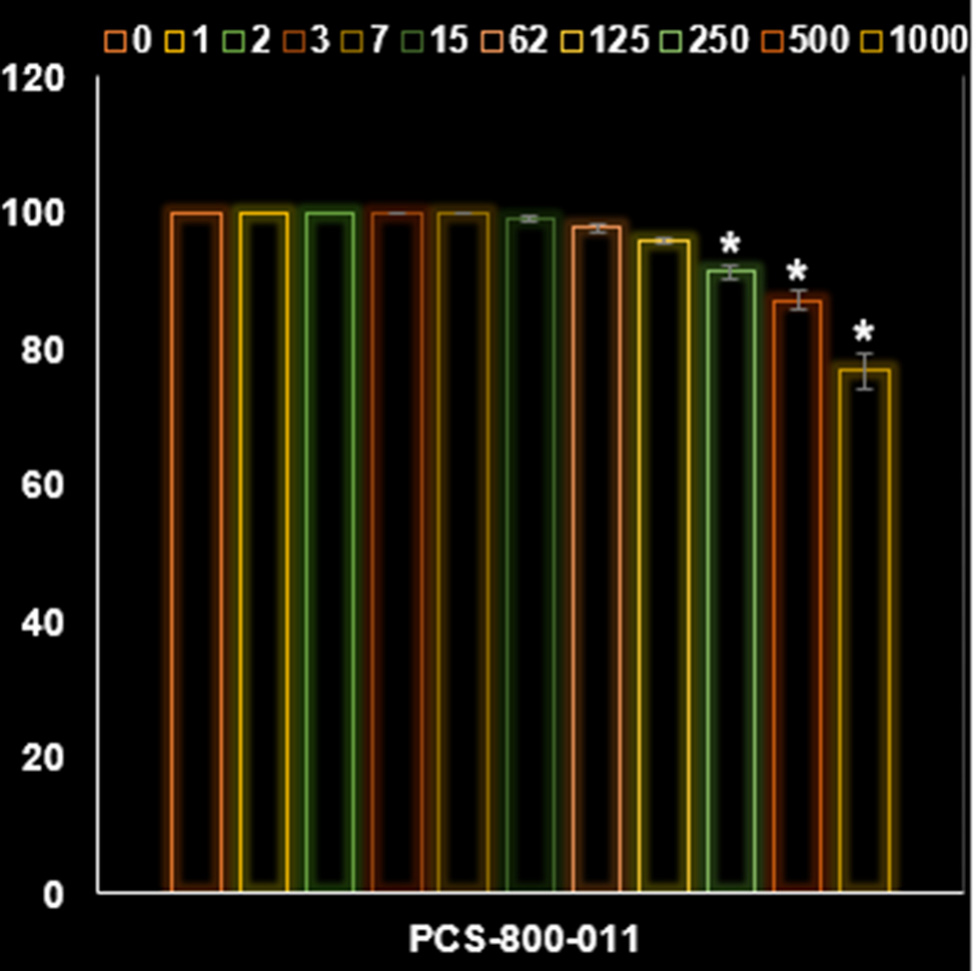
Activities of AgNPs@*S. aromaticum* at different concentrations (0–1,000 µg/mL) on the PCS-800-011 primary peripheral blood mononuclear cells viability (%).

**Figure 9:**
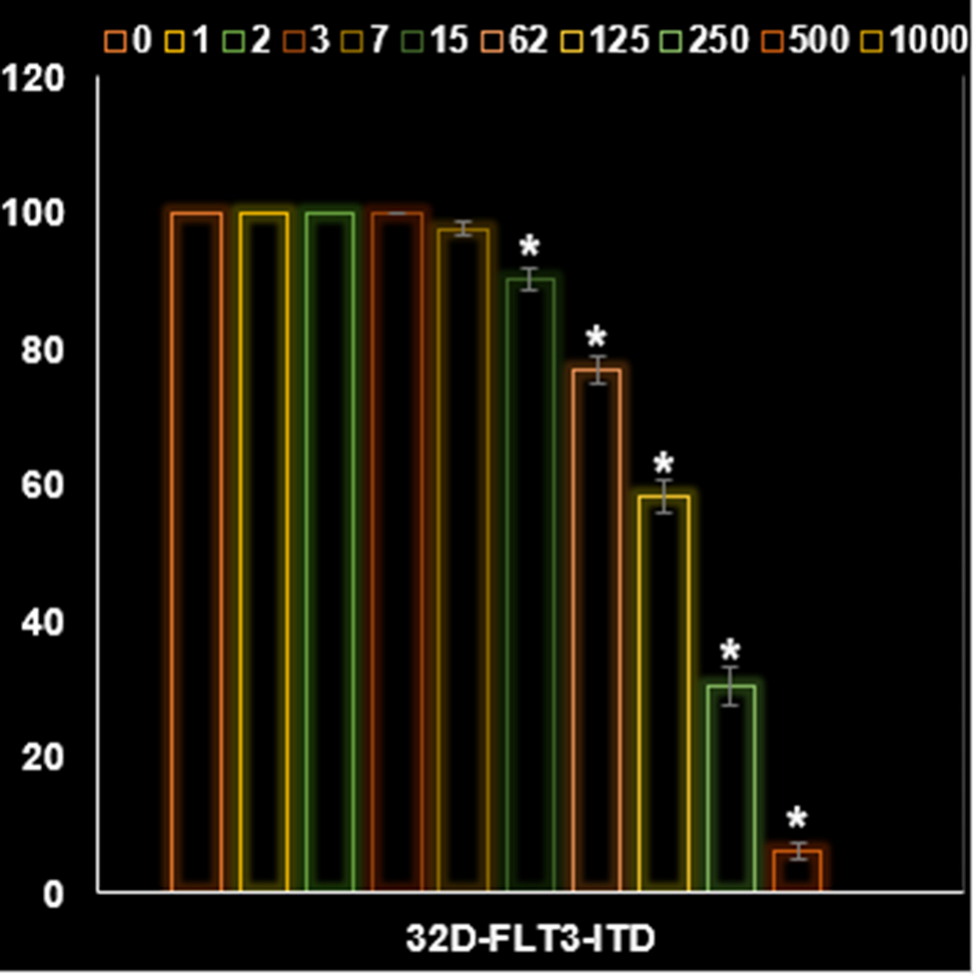
Activities of AgNPs@*S. aromaticum* at different concentrations (0–1,000 µg/mL) on the leukemia 32D-FLT3-ITD cell viability (%).

In this investigation, AgNPs appear to exhibit anticancer activity on 32D-FLT3-ITD human leukemia cells due to their antioxidant properties. As shown in Figures 8 and 9, AgNPs significantly decreased the viability of cancer cells, partculary the 32D-FLT3-ITD human leukemia cell line. Figure 9 demonstrates that the IC_50_ value of the NPs against 32D-FLT3-ITD human leukemia cells was 162 µg/mL. Despite the aggregation of NPs which resulted from the FE-SEM image the AgNPs revealed acceptable anti-cancer activity against the leukemia cells.

**Figure 10:**
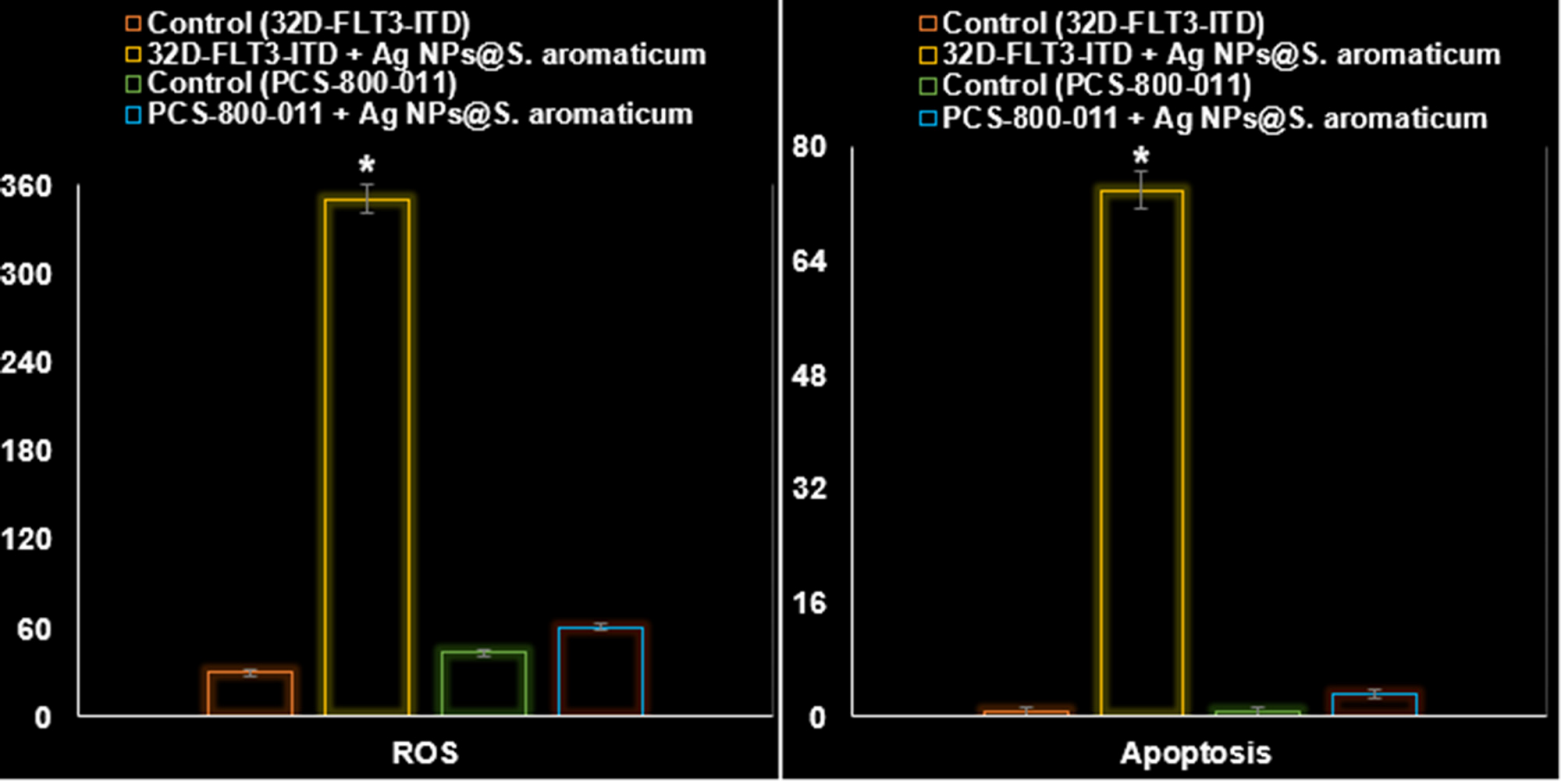
Effects of AgNPs@*S. aromaticum* on the PCS-800-011 and 32D-FLT3-ITD cell line’s ROS (a.u.) and apoptosis (%).

**Figure 11:**
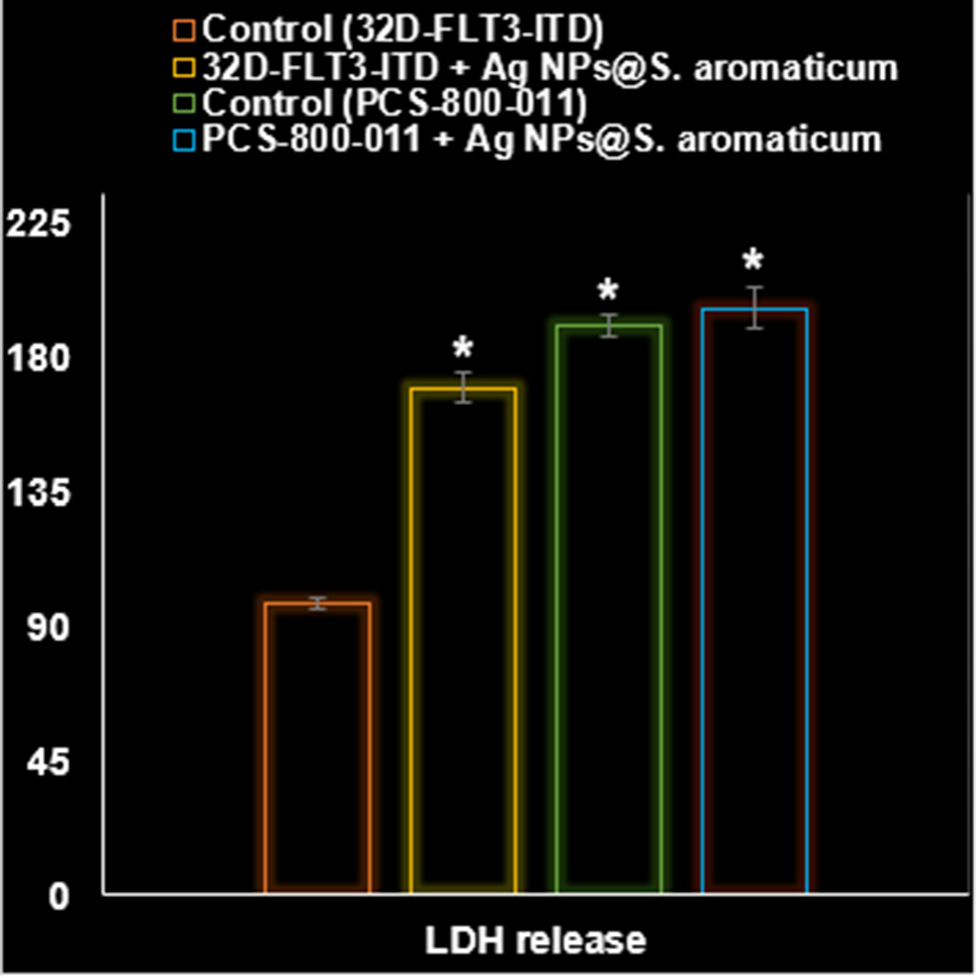
Effects of AgNPs@*S. aromaticum* on the PCS-800-011 and 32D-FLT3-ITD cell line’s LDH release (%).

**Figure 12:**
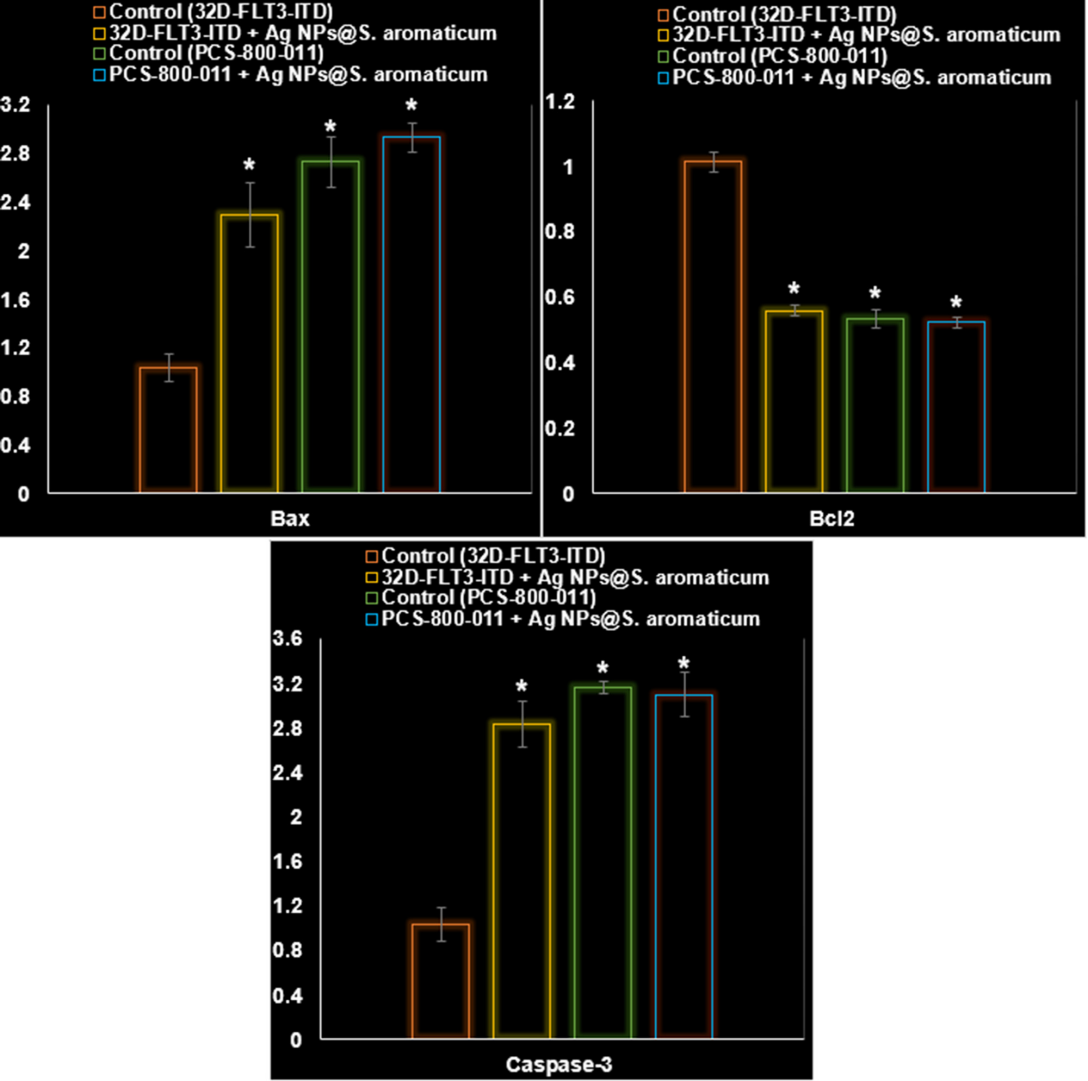
Effects of AgNPs@*S. aromaticum* on the PCS-800-011 and 32D-FLT3-ITD cell line’s Bax, Bcl2, and Caspase-3 mRNA expression levels (fold exchange of mRNA).

**Figure 13:**
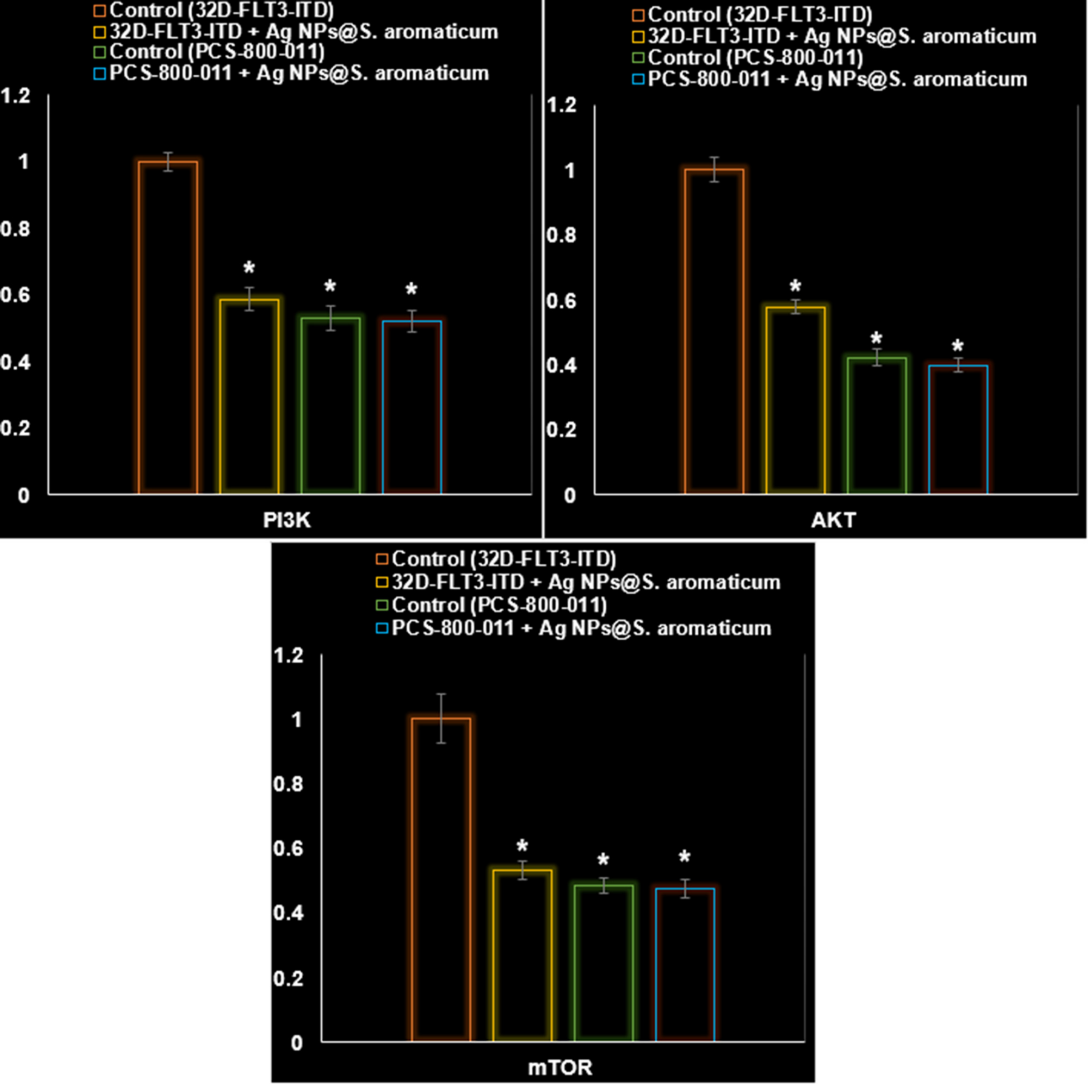
Characteristics of AgNPs@*S. aromaticum* on the control of the PCS-800-011 and 32D-FLT3-ITD cell line’s PI3K/AKT/mTOR signaling pathway (fold exchange of mRNA).

In addition to significantly impacting the PI3K/AKT/mTOR pathway within 32D-FLT3-ITD human leukemia cells (*P* ≤ 0.01), AgNPs@*S. aromaticum* also significantly increased the levels of ROS, apoptosis, and LDH release (*P* ≤ 0.01). Figures 10–13 provide evidence that AgNPs@*S. aromaticum* exhibits protective properties against 32D-FLT3-ITD human leukemia *in vitro*. Recent molecular data further demonstrates that AgNPs@*S. aromaticum* significantly upregulated the levels of Caspase-3 and Bax mRNA expression (*P* ≤ 0.01), while downregulating Bcl2 mRNA expression in 32D-FLT3-ITD human leukemia cells (*P* ≤ 0.01) (Figure 12). Additionally, AgNPs@*S. aromaticum* significantly decreased the fold change in mRNA expression of PI3K, AKT, and mTOR compared to the control group 32D-FLT3-ITD human leukemia cells (*P* ≤ 0.01) (Figure 13). No significant difference between the Control (PCS-800-011) and PCS-800-011 + AgNPs@*S. aromaticum* groups in the above parameters (*P* ≤ 0.01) (Figures 10–13).

The findings from our investigation align with the research conducted by Gu et al. [45], who reported an IC_50_ value of 27.45 μg/mL after 24 h of incubation. The difference in incubation duration could be related to the utilization of artificially synthesized NPs in their study, as opposed to our naturally derived NPs. However, our findings contrast significantly with those of Alipour et al. [46]. Their research focused on examining the cytotoxic impact of NPs on SKOV3 cell lines, revealing an IC_50_ value of 8.05 μM after 72 h. The variation in results could be attributed to the utilization of distinct methods for assessing cytotoxicity (MTT assay versus SRB assay), in addition to the potential influence of naturally sourced NPs. The findings indicated a notable enhancement in response to Cisplatin in SKOV3 cells upon treatment with NPs. According to the current investigation, it is evident that NPs derived from natural sources exhibit great potential as an effective anticancer agent specifically for breast cancer. NPs exhibited a noticeable cytotoxic effect on 32D-FLT3-ITD cells, along with a definite concentration–response relationship (Figures 8–13).

The utilization of rod-shaped nanostructures for remedial purposes is supported by the promising findings of this study, as well as the ongoing advancements in green nanotechnology [47]. Previous studies have reported that NPs induce proteotoxic stress and acute oxidative stress in ovarian carcinoma cells, leading to their death and apoptosis [47,48]. The primary mechanism of toxicity from NP exposure is the production of ROS through oxidative stress. Increased levels of ROS serve as triggers for apoptosis. NPs cause a significant increase in the levels of cell cycle checkpoint proteins p53, caspase-3, and Bax, while simultaneously decreasing the expression of the antiapoptotic protein Bcl-2 [48]. Additionally, it has been observed that NPs have the ability to induce apoptosis through intrinsic mitochondrial pathways. They can initiate apoptosis by decreasing the potential of the mitochondrial membrane and, on the other hand, increasing the ratio of Bax to Bcl2 [49]. The HA/ZnO nanocomposite, synthesized using green methods, shows promising potential as a highly effective cancer therapy [50]. According to previous studies, AgNPs induce apoptosis in leukemia cells primarily through the generation of ROS, leading to oxidative stress, DNA damage, and mitochondrial dysfunction [51]. Studies have shown that AgNPs inhibit the viability of acute myeloid leukemia cells by increasing ROS production, which triggers the intrinsic mitochondrial apoptotic pathway characterized by cytochrome c release, activation of caspase-9 and caspase-3, and modulation of Bcl-2 family proteins with increased pro-apoptotic Bax and decreased anti-apoptotic Bcl-2 expression [51–53]. In chronic lymphocytic leukemia cells, AgNPs cause mitochondrial membrane depolarization and calcium dysregulation, further promoting apoptosis [53–55]. Additionally, AgNPs can sensitize leukemia cells to chemotherapeutic agents, such as 4-HPR, enhancing apoptotic cell death. These effects are supported by evidence of DNA repair gene modulation, such as increased MLH1 expression, which contributes to apoptosis. Overall, AgNPs induce leukemia cell apoptosis via oxidative stress-mediated mitochondrial pathways, caspase activation, and gene regulation, making them promising candidates for leukemia therapy [52,53].

## 4 Conclusion

In conclusion, an aqueous extract of *S. aromaticum* was utilized to environmentally friendly manufacture AgNPs. The applied analytical techniques confirmed the synthesis of AgNPs. The TEM and FE-SEM images revealed a spherical morphology for AgNPs with a tendency to aggregate, and the average size of AgNPs was less than 50 nm. Results from UV–Vis and FT-IR spectrophotometers confirmed the formation of AgNPs. The effectiveness against leukemia cells decreased as the concentration of AgNPs@*S. aromaticum* increased. There was a dose-dependent correlation between leukemia cell survival and AgNPs@*S. aromaticum*, indicating anti-leukemia properties. The findings suggest that following further human clinical studies, AgNPs@*S. aromaticum* may have application in the biomedical industry.
